# Long-Term Neurophysiological Outcomes in Patients Undergoing Coronary Artery Bypass Grafting

**DOI:** 10.21470/1678-9741-2020-0390

**Published:** 2021

**Authors:** Irina V. Tarasova, Olga A. Trubnikova, Irina D. Syrova, Olga L. Barbarash

**Affiliations:** 1 Research Institute for Complex Issues of Cardiovascular Diseases, Kemerovo, Russia.

**Keywords:** Cognitive Dysfunction, Coronary Artery Bypass, Alpha Rhythm, Neurophysiological Tests, Postoperative Cognitive Complications

## Abstract

**Introduction:**

This study aims to evaluate late postoperative neurophysiological outcomes in patients after coronary artery bypass grafting (CABG).

**Methods:**

Forty-five male patients with stable coronary artery disease aged 45-69 years underwent extended neuropsychological assessment using the software Status PF and electroencephalographical examination 3-5 days before CABG and 5-7 years after CABG. Postoperative decline in cognitive functions was determined by a 20% decrease in the cognitive indicator compared to that at baseline on 20% of the tests included in the Status PF battery. Statistical analysis was performed using the software STATISTICA 10.0. Multiple regression was used to identify demographic, clinical, and electroencephalographical variables associated with adverse cognitive outcomes.

**Results:**

Cognitive decline was observed in 54% of the patients in the long-term postoperative period. Five to seven years after CABG, all patients have shown an increase in the theta rhythm power compared to the preoperative values, which is most pronounced in the frontal and temporal areas of the right hemisphere (*P*=0.04), along with a decrease in the alpha rhythm in the posterior areas of the cortex (*P*=0.005). Multiple regression has reported that the main predictors of cognitive impairment are slower mean alpha frequency, decreased theta-2 rhythm with eyes closed in the right temporal area, and increased theta-2 rhythm with eyes open in the left temporal area (F(5.39)=8.81; *P*<0.00007; adjusted R-squared=0.57).

**Conclusion:**

Our findings indicate that 54% of the patients suffer from postoperative cognitive decline associated with increased theta and decreased alpha rhythms 5-7 years after CABG.

**Table:** 

Abbreviations, acronyms & symbols				
ACEi	= Angiotensin-converting enzyme inhibitor		IAF	= Individual alpha frequency
ANOVA	= Analysis of variance			
BDI-II	= Beck Depression Inventory II		Me	= Median
CABG	= Coronary artery bypass grafting		MMSE	= Mini-Mental State Examination
CAD	= Coronary artery disease		MSCT	= Multi-slice spiral computed tomography
CCB	= Calcium channel blockers		NYHA	= New York Heart Association
CSI	= Cognitive status index		POCD	= Postoperative cognitive dysfunction
EEG	= Electroencephalography		S.E.	= Standard error
FAB	= Frontal Assessment Battery		USA	= United States of America

## INTRODUCTION

Coronary artery disease (CAD) remains the leading cause of death worldwide. Cognitive impairment is common in patients with CAD. These patients are the most difficult ones to treat, particularly those who undergo cardiac surgery ^[1]^. Postoperative cognitive dysfunction (POCD) is a decline in cognitive functions following surgery, characterized by impairment of attention, concentration, and memory that may have long-term implications ^[2]^. Recent studies have demonstrated that early POCD occurs in almost 70% of patients undergoing coronary artery bypass grafting (CABG). One year after surgery, cognitive decline occurs in 30-50% of cases, affecting their long-term prognosis ^[1,3]^. Recent studies have reported that cognitive disorders may progress from mild cognitive impairment to dementia during a five-year period ^[4,5]^. Persistent POCD affects the quality of life, leading to disability and social maladjustment ^[5,6]^. Moreover, it is associated with an increased risk of mortality during a seven-year follow-up ^[6]^.

The neurophysiological mechanisms underlying the development of POCD are still not clear. A significant proportion of CABG patients are middle-aged or older adults, who suffer from chronic brain ischemia and are more vulnerable to cognitive decline after surgery ^[1]^. In addition, surgically-induced brain damage may contribute to the cumulative brain atrophy ^[7]^.

The detection of minimal or subclinical signs of brain dysfunction following CABG is still under debate. Quantitative electroencephalography (EEG) is commonly used for the assessment of both normal brain functioning and the development of brain pathology ^[8-11]^. Prior studies have shown the relationship between EEG abnormalities and cognitive impairment ^[8,10,11]^. A slowing down of resting-state EEG has been reported to be a marker of cortical dysfunction ^[3,10,11]^. Bonanni et al. ^[11]^ have found that patients with Parkinson’s disease experiencing cognitive impairment and Lewy body dementia demonstrate higher power values of theta activity than healthy control subjects. However, little is known about EEG changes in patients after on-pump CABG ^[3]^. The long-term neurophysiological effects following surgical myocardial revascularization are poorly understood. Moreover, little is known about the structure of cognitive impairment during the long-term postoperative period and the corresponding functional activity of the brain. To facilitate and improve the diagnostic accuracy, detailed neuropsychological examination using the multichannel digital EEG and psychometric tests may be used to detect the long-term brain changes associated with postoperative cognitive impairment.

Therefore, our study aims to evaluate the neurophysiological outcomes of patients 5-7 years after CABG.

## METHODS

### Participants

Forty-five consecutive patients with CAD were recruited to this prospective study in the period from 2010 to 2012. This study was conducted in accordance with the principles of the Helsinki Declaration and was approved by the Institute’s Local Ethics Committee (protocol number nº 23 issued on February 02, 2011). The inclusion criteria were as follows: male sex, aged 45-70 years, undergoing elective on-pump CABG, and obtained written informed consent to participate in the study. The exclusion criteria were as follows: diagnosis of neurological or psychiatric disorders that may interfere with cognition; patients with severe depression (score of ≥ 8 on the Beck Depression Inventory) ^[12]^; severe comorbidities; abuse of alcohol or psychotropic drugs; seriously reduced eyesight or other sensory deficits that could affect the assessment of cognitive status; and Mini-Mental State Examination (MMSE) score of ≤ 24 and Frontal Assessment Battery (FAB) score of ≤ 11 ^[13,14]^. Women and patients older than 70 years were excluded from the study in order to avoid the potential effect of the gender and age on the results of the study.

### Clinical Assessment

Patients underwent general medical, neurological, and instrumental examination before CABG and 5-7 years after CABG (January 2016 to December 2017). The examiners were blinded to the patients’ cognitive statuses. The severity of coronary artery lesions before CABG was assessed using coronary angiography (Innova 3100; GE Medical Systems, Carrollton, Texas, United States of America [USA]) and the SYNTAX Score calculator (http://www.syntaxscore.com/calculator/start.htm) ^[15]^. Carotid artery ultrasound and echocardiography were performed with the Vivid 7 ultrasound machine (GE Medical Systems). Multi-slice spiral computed tomography (MSCT) was performed with the SIEMENS SOMATOM Sensation 64 (Germany) to detect any nervous system abnormalities. The width of the third ventricle and the ventriculocranial index (Evans index) were measured. Any occurrence of leukoaraiosis, cysts, and gliosis was recorded.

The pre and postoperative clinical and demographic data of patients and prescribed medical therapy are presented in Table 1.

**Table 1:** Clinical and demographic characteristics of patients before CABG and 5-7 years after CABG.

Variable	Patients, n=45	
	Before CABG	5-7 years after CABG
Age, years, Me (Q25; Q75)	56.0 (52; 59)	62.5 (58,5; 66)
**Educational attainment, n (%)** 8 years 10-12 years 15 years	2 (4%) 31 (69%) 12 (27%)	-
Left ventricular ejection fraction, %, Me (Q25; Q75)	60.0 (52; 63)	60.5 (54; 63)
Functional class angina, n (%) 0 I-II III	0 (0%) 30 (77%) 15 (33%)	32 (71%) 12 (27%) 1 (2%)
**Functional class NYHA, n (%)** I-II III	37 (82%) 8 (18%)	44 (98%) 1 (2%)
Severity of coronary arteries lesions by the SYNTAX scale before surgery, scores, Me (Q25; Q75)	22.5 (16.5; 29.3)	-
History of myocardial infarction, n (%)	6 (13%)	-
Myocardial infarction after surgery, n (%)	-	1 (2%)
Stenoses of the carotid arteries, n (%)	21 (47%)	23 (51%)
Diabetes mellitus Type 2, n (%)	10 (22%)	10 (22%)
**Cardiac rate disturbance and disorder of conduction, n (%)** Atrial fibrillation Ventricular contractions, III-IV Lown grade	0 (0%) 0 (0%)	4 (9%) 7 (16%)
**Medication, n (%)** ACEi Statin Beta-blockers Antiplatelet drugs CCB Nitrates	40 (89%) 30 (67%) 44 (98%) 43 (96%) 19 (42%) 7 (16%)	20 (44%) 31 (69%) 37 (82%) 42 (93%) 0 (0%) 1 (2,2%)
MMSE, scores, Me (Q25; Q75)	28 (26; 28)	28 (27; 29)
FAB, scores, Me (Q25; Q75)	17 (16; 17)	16 (15; 17)
BDI-II, scores, Me (Q25; Q75)	2 (1; 3)	3 (2; 5)

ACEi=angiotensin-converting enzyme inhibitor; BDI-II=Beck Depression Inventory II; CABG=coronary artery bypass grafting; CCB=calcium channel blockers; FAB=Frontal Assessment Battery; Me=median; MMSE=Mini-Mental State Examination; NYHA=New York Heart Association

### Surgical Procedure

On-pump CABG was performed under standard perfusion conditions with normothermia and intravenous anesthesia (propofol). The mean cardiopulmonary bypass time was 100.2±28.24 min, and the aortic cross-clamping time was 62.8±16.86 min. The number of grafts was 2.6±0.71. Invasive hemodynamic control was performed throughout the surgery. Neither episodes of hypotension, nor oxygenation impairment were observed according to the cerebral oximetry (INVOS-3100, SOMANETICS, USA).

### Neuropsychological Assessment

MMSE and FAB were used for cognitive screening. Neuropsychological testing included the assessment of psychomotor and executive function, attention, and short-term memory using the neuropsychological test battery from the psychophysiological complex software Status PF. The detailed description of the neuropsychological tests is shown in Table 2. Postoperative cognitive decline five years after CABG was determined by a 20% decrease in the cognitive score compared to baseline in 20% of the tests ^[16,17]^. The integral cognitive status index (CSI) was calculated by determining the average distance from the patient’s values to the reference ones using the following formula:


$$\mathrm{CSL}=1-\frac{\sqrt{{\left(1-Y1\right)}^{2}+{\left(1-Y2\right)}^{2}+{\left(1-Y3\right)}^{2}+{\left(1-Y4\right)}^{2}+{\left(1-Y5\right)}^{2}}}{5}$$

**Table 2:** Cognitive test battery for assessing cognitive function in CABG patients.

Cognitive tests and indicators	Description of the procedure
**Complex visual-motor reaction** Reaction time, ms Errors, n	Reaction latencies of the right and left hands to stimuli (different colors of rectangles) when the subject should choose one of the three presented signals (the number of signals in the test is 30)
**Level of functional mobility of nervous processes responses to "feedback"** Reaction time, ms Errors, n Missed signals, n	The previous test is conducted in the feedback mode. The duration of the exposure to the test signal (see above) is changed automatically; the exposure of the next signal is shortened by 20 ms with each correct answer and extended by 20 ms, if the answer is wrong (the number of signals in the test is 120)
**Performance of the brain responses to "feedback"** Reaction time, ms Errors, n Missed signals, n	The previous test is conducted in the feedback mode for a fixed period (5 min). It is necessary to process the maximum number of signals presented with a given exposure
**The Bourdon's test** Processed symbols per 1 min, n Processed symbols per 4 mins, n	The subject is provided with the alphabetic version of the Bourdon's test to highlight certain letters for the lead time of 4 mins
10 words memorizing test, n	To remember as many of 10 words presented one after another as possible
10 numbers memorizing test, n	To remember as many of 10 numbers presented one after another as possible
10 nonsense syllable memorizing test, n	To remember as many of 10 nonsense syllables presented one after another as possible

CABG=coronary artery bypass grafting

where:

Y is a recording value of the cognitive indicator; Y1 is the mean value of the reaction time in psychomotor and executive function tests; Y2 is the mean value of the errors in psychomotor and executive function tests; Y3 is the mean value of the missing signals in psychomotor and executive function tests; Y4 is the mean value of the short-term memory indicator; and Y5 is the mean value of the attention indicator.

### EEG Recording

A monopolar EEG in 62 channels (bandwidth 0.1-50.0 Hz), in accordance with the international 10-20 system, with the eyes closed and open was recorded with an amplifier (Neuvo SynAmps2, Compumedics, Charlotte, North Carolina, USA) using a modified 64-channel cap (QuikCap; Neurosoft, El Paso, Texas, USA). The reference electrode was attached to the tip of the nose, and the ground electrode to the forehead (impedance < 5 kΩ). The recording duration was 10 min (five minutes with the eyes closed). Data were analyzed offline, and a visual search for recording artifacts was conducted. Artifact-free EEG fragments were divided into 2-s epochs and subjected to the Fourier transform. The peak individual alpha frequency (IAF) for each patient was determined. Because the mean IAF of the study group was 9.4 Hz, we used standard frequency bands. For each patient, the EEG power values were averaged within theta-1 (4-6 Hz), theta-2 (6-8 Hz), alpha-1 (8-10 Hz), alpha-2 (10 -13 Hz), beta-1 (13-20 Hz), and beta-2 (20-30 Hz) ranges. Further, the EEG power values recorded at nearby electrode sites were summarized in five areas in the left and right hemispheres:

1. Frontal (Fp1/2+AF3/4+F1/2+Fp3/4+Fp5/6+F7/8);
2. Central (FC1/2+FC3/4+FC5/6+C1/2+C3/4+C5/6);
3. Temporal (FT7/8+T7/8+TP7/8);
4. Parietal (CP1/2+CP3/4+CP5/6+P1/2+P3/4+P5/6+P7/8);
5. Occipital (PO3/4+PO5/6+PO7/8+O1/2).

The parameters recorded at the midline (Fpz, Fz, etc.) were excluded from the analysis.

#### Statistical Analyses

Statistical analysis was performed using the software STATISTICA 10.0 (StatSoft, Tulsa, Oklahoma, USA). Variable distributions were examined using scatterplots/histograms and normality tests. Continuous variables are expressed as medians and quartiles, and categorical variables are expressed as numbers and percentages. Two-tailed Mann-Whitney U and Wilcoxon tests were used for continuous variables. Continuity-corrected χ^2^ or Kruskal-Wallis tests were used for categorical variables. Non-normally distributed EEG power values were log-transformed. Repeated-measures analyses of variance (ANOVAs) were used to analyze EEG power for the abovementioned frequency ranges and for the eyes-open and eyes-closed resting states, with GROUP (patients with and without postoperative cognitive decline), TIME OF STUDY (before CABG, five years after CABG), AREA (frontal, central, temporal, parietal, occipital), and LATERALITY (left and right hemispheres) as within-subject factors. The Greenhouse-Geyser correction of statistical significance was performed. A subsequent statistical significance analysis was performed using post-hoc and Newman-Kels tests. The significance level was 5%.

To identify correlations between EEG and neuropsychological test data, the Pearson’s correlation analysis was performed. To correct the multiplicity of comparisons, the significance level was increased up to *P*≤0.01. To identify demographic, clinical, and EEG variables associated with adverse cognitive outcomes, the multiple linear regression with a stepwise approach was applied. Independent variable selection was based on our previous research on POCD in CAD patients, the characteristics of the study population, and the correlation analysis data described ^[1,3]^. CSI was the dependent variable.

## RESULTS

### Clinical and Neuropsychological Data

There were no significant adverse cardiovascular events among patients in the early and long-term postoperative period of CABG (the mean observation period was 6.5 years). One patient had myocardial infarction and underwent percutaneous coronary intervention. The details are provided in Table 1.

Serial MSCT reported specific morphological changes in the brain tissue. The third ventricle significantly expanded from 7.1±2.0 mm to 8.6 ±2.4 mm (*P*=0.04). The number of patients with cysts and gliosis (3 [6.7%] *vs.*. 12 [26.7%], *P*=0.0001) and the number of patients with leukoaraiosis (13 [29%] *vs.* 36 [81%], *P*=0.0001) increased in the long-term period. The Evans index did not change significantly (29 *vs.* 31, *P*=0.8).

The incidence of late postoperative cognitive decline was 54% (24 patients). The structure of postoperative cognitive decline consisted of impaired neurodynamic function (70%, 30 patients), memory (47%, 21 patients), and attention disorders (21%, nine patients). The number of patients without impairment was 9% (four patients).

### EEG Data Analysis

The ANOVA revealed significant interactions associated with postoperative cognitive decline in the theta and alpha frequency ranges.

**Fig. 1:**
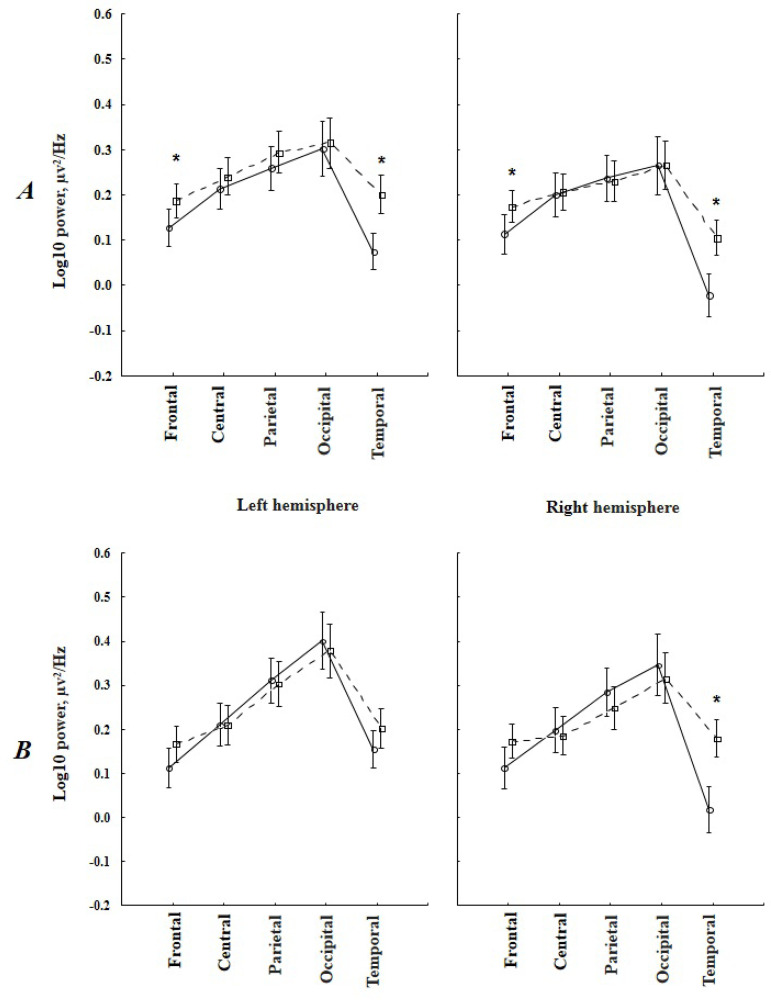
Topographic changes of the theta-1 rhythm power with eyes closed in patients with cognitive decline (А) and without cognitive decline (B) in the long-term period after coronary artery bypass grafting. Solid lines indicate preoperative indicators, dashed lines indicate 5-7 years after surgery. *marked significant differences (P≤0.05).

For the theta-1 power values with the eyes closed, the following significant interactions were found: TIME × AREA - (F_(4, 172)_=13.87, *P*=0.000), TIME × AREA × LATERALITY - (F_(4, 172)_=6.56, *P*=0.0005), and GROUP × TIME × AREA × LATERALITY - (F_(4, 172)_=2.89, *P*=0.04). Five to seven years after CABG, patients demonstrated a significant increase in the theta-1 power compared to baseline in the frontal and temporal areas, with more pronounced changes in the right hemisphere. Baseline and postoperative theta-1 power did not differ between the groups. However, patients with postoperative cognitive decline exhibited significantly increased theta-1 power in the frontal and temporal left and right hemispheres, whereas patients without cognitive decline demonstrated its increase in the temporal regions of the right hemisphere (Figure 1).

**Fig. 2:**
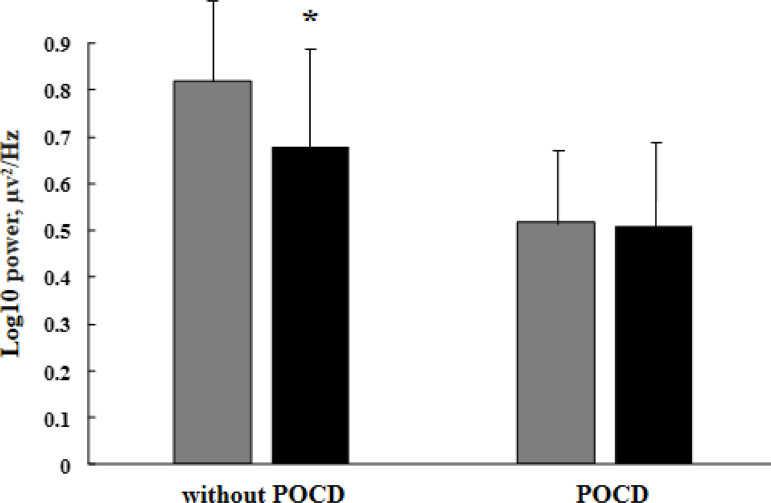
Changes of alpha-1 rhythm power with eyes closed in the left hemisphere in patients depending on the cognitive decline in the long-term period after coronary artery bypass grafting. Gray columns indicate preoperative indicators, black columns indicate 5-7 years after surgery. * marked significant differences (P≤0.05). POCD=postoperative cognitive dysfunction

For the alpha-1 range, with the eyes closed, the following significant interactions were found: TIME × AREA - F_(4, 172)_=5.62, *P*=0.005) and GROUP × TIME × LATERALITY - (F_(1, 43)_=4.80, *P*=0.03). All patients demonstrated a decrease in the alpha-1 power compared to baseline in the parietal and occipital cortices. Between-group differences were found in the left hemisphere. Before surgery, the alpha-1 power was higher in the group of patients without cognitive decline compared to patients with postoperative cognitive decline. At the follow-up, only patients without cognitive decline demonstrated a significant decrease in the alpha-1 power in the left hemisphere (Figure 2). There were no significant changes found for the right hemisphere.

For the alpha-1 range, with the eyes open, the factor of TIME - (F_(1, 43)_=4.65, *P*=0.04) interacted with TIME × AREA - (F_(4, 172)_=4.96, *P*=0.006) and GROUP × TIME × AREA × LATERALITY - (F_(4, 172)_=5.07, *P*=0.0025). All patients exhibited increased alpha-1 power compared to baseline. Significant changes were found in the occipital and temporal cortices, which were more pronounced in the group of patients with cognitive decline.

### Correlation Analysis

**Fig. 3:**
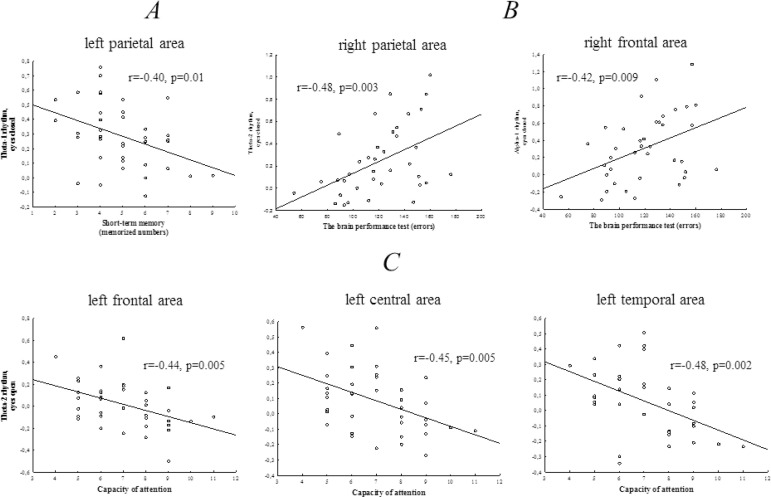
Correlations between the cognitive status indicators and electroencephalographical power: (A) a correlation between number of memorized digit and log-transformed theta-1 rhythm power values (eyes closed); (B) correlations between errors in the brain performance test and log-transformed theta-2 and alpha-1 rhythms power values (eyes closed); and (C) correlations between a capacity of attention and log-transformed theta-2 rhythm power values (eyes open) in the coronary artery disease patients.

The results of the correlation analysis are presented in Figure 3. The number of memorized numbers negatively correlated with theta-1 power with the eyes closed in the left parietal cortex. The number of errors during the brain performance test directly correlated with the theta-2 and alpha-1 rhythm power with the eyes closed in the right hemisphere. In addition, the number of missed signals negatively correlated with the theta-2 power in the posterior brain regions (left and right hemispheres). Negative correlations between the attention and theta-2 power with the eyes open in all areas of the left hemisphere were found.

### Multiple Regression Analyses

The first multiple regression model was generated based on clinical and demographic variables. We found that the time of cardiopulmonary bypass had the highest prognostic weight (Beta coefficient=-0.35; *P*=0.0003). The estimated regression standardized coefficients were as follows: *P*=0.41; R-squared=0.17; adjusted R-squared=0.09; F_(3.41)_=2.28; *P*=0.09. The standard estimation error was 0.137. However, this model had a low prognostic value.

The next regression model was generated with a compilation of clinical variables and EEG data. The theta-2 power had the greatest prognostic weight along with the mean alpha frequency (Table 3). The estimated regression standardized coefficients of the model were as follows: R=0.80; R-squared=0.64; adjusted R-squared=0.57; F_(5.39)_=8.81; *P*<0.00007. The standard estimation error was 0.09. Slower mean alpha frequency, decreased theta-2 rhythm with the eyes closed in the right temporal area and sequential organ failure assessment score, increased theta-2 rhythm with the eyes open in the left temporal area and theta-2 rhythm with the eyes closed in the right frontal area were the independent predictors of cognitive decline. These predictors explained 57% of the variance of the CSI scores.

**Table 3:** Results of the regression analysis of factors associated with postoperative cognitive decline five years after CABG in CAD patients.

Dependent variable - integral cognitive status index (or CSI)	Beta	S.E. - Beta	t	*P*-value
Intercept			-2.335	0.028
Mean alpha frequency	0.669	0.133	5.025	0.00004
Theta-2 rhythm with eyes closed in right temporal area	1.134	0.258	4.394	0.0002
Theta-2 rhythm with eyes open in left temporal area	-0.600	0.162	-3.711	0.001
Sequential organ failure assessment score	0.296	0.131	2.268	0.03
Theta-2 rhythm with eyes closed in right frontal area	-0.294	0.219	-1.338	0.19

CABG=coronary artery bypass grafting; CAD=coronary artery disease; S.E.=standard error

## DISCUSSION

We found that 54% of patients demonstrated a decline in cognitive function compared to baseline during the long-term postoperative period of CABG (5-7 years).

The mechanisms underlying the development of POCD are still poorly understood. Some researchers have reported that cerebral microembolic load, inflammatory response, and non-physiological perfusion of cardiopulmonary bypass contribute to postoperative cognitive impairment in early postoperative period and late cognitive recovery ^[2,17]^. However, Selnes et al. ^[16]^ have proposed that preexisting cerebrovascular disorders more likely contribute to long-term POCD rather than cardiopulmonary bypass.

Our data have shown that long-term postoperative cognitive decline is most pronounced in the neurodynamic domains and short-term memory. Notably, neurodynamic test performance allows evaluating executive control. Alosco et al. ^[18]^ have found that patients with cardiovascular diseases exhibit decreased memory and executive functions. Patients with cardiovascular diseases are highly susceptible to ischemic changes in the frontal brain regions, resulting in executive function impairment ^[19,20]^. Additionally, short-term memory decline has been found in patients with heart failure ^[21,22]^. Our findings are consistent with the abovementioned studies, but these neurophysiological results have been obtained in a specific sample of patients undergoing CABG. The factors associated with cardiac surgery affect brain functions, and their effects are particularly pronounced in the early postoperative CABG period. However, less is known about their long-term impact. Researchers have suggested that brain damage in the early postoperative CABG may underlie the development of persistent cognitive deficit ^[1,2,6,17]^.

We have found that cognitive deficit is accompanied with the morphological changes in brain tissue and an increase in the background theta activity with the eyes closed and alpha activity with the eyes open in the long-term period after CABG. The resting-state alpha activity with closed eyes has decreased compared to preoperative indicators. Previous studies have shown that increased lower frequencies, reduced complex activities, and incoherent cortical regions/fast rhythms recorded on the electroencephalogram may indicate POCD ^[3,23]^. We may suggest that CABG patients suffer from insufficient cerebral circulation, causing neuronal dysfunction, in late postoperative period. As a result, a negative shift of the EEG rhythms occurs. Kramberger et al. ^[24]^ demonstrated that the dominant background EEG activity slowing is correlated with an increased T-tau protein and a lower Aβ42/P-tau ratio in cerebrospinal fluid. The atrophic changes in the brain, such as an expansion of the brain ventricular system and leukoaraiosis, can be regarded as clinical manifestations of cerebral microangiopathy ^[25]^. In our study, almost 50% of patients suffered from carotid stenosis and 9% had postoperative atrial fibrillation. These comorbidities could promote pathologic cardiac remodeling and contribute to the brain ischemia.

Pathological rearrangements of brain oscillations are associated with altered regulation, reduced-complexity oscillatory processes, and dysfunctions of neurotransmission ^[26]^. It has also been hypothesized that the diminished predominance of posterior alpha activity over slow activity (theta) and an increase in theta activity relative to alpha activity may be associated with impaired regulation of the cholinergic basal forebrain. This could cause prolonged agitation in the stem cholinergic pathways ^[8]^.

Thus, the combination of EEG resting-state characteristics can indicate an imbalance between cortical and subcortical structures, and a decrease in the functional activity of the cortex ^[27,28]^. Topographically identified changes in the background EEG activity were localized within the frontal, temporal, and parieto-occipital regions. The results of the correlation and multiple regression analyses confirmed the involvement of these brain structures in the process of cognitive decline in the late postoperative period. Thus, the poorest short-term memory values corresponded to large values of background theta-1 activity with the eyes closed in the left parietal cortex. Neurodynamic disorders were associated with impaired function of the right parietal-occipital regions of the cortex. Neurodegeneration primarily affects the hippocampus and adjacent brain regions, i.e., the cingulate and parietal-temporal cortex ^[29,30]^. Further, the recent studies of cognitive disorders in the cohort of elderly patients with cardiovascular diseases have indicated certain difficulties in the differentiation of the neurodegenerative and ischemic patterns of brain damage; rather, we should refer to a mixed etiology of cognitive deficit ^[19,31]^.

The data obtained in this study confirm this assumption. Since EEG signs of cerebral dysfunction in the late period of CABG are localized in the frontal and parieto-temporal brain regions, cognitive decline of executive functions and short-term memory is found. However, the factors contributing to late cognitive decline after CABG remain unclear. The high incidence of cognitive decline in the long-term postoperative period and the ambiguity of the underlying mechanisms prompt further study of this phenomenon in specific groups of CAD patients. Our findings indicate the need to improve approaches to the postoperative follow-up management of patients undergoing cardiac surgery, capable of minimizing the development of adverse neurological outcomes.

### Limitations

The findings of our study should be interpreted within the context of its limitations. We did not recruit a control group of healthy volunteers to assess age-related changes in this group of patients. We have a small sample of patients (n=45), since we were recruiting only consecutive ones. Additionally, only male CAD patients were included. Therefore, it is unclear whether our findings can be extrapolated to women. We plan to address all of these issues in our future studies.

## CONCLUSION

Our findings demonstrate that 54% of the patients suffer from a postoperative cognitive decline associated with increased theta and decreased alpha rhythms 5-7 years after CABG. The long-term postoperative cognitive decline is most pronounced in the neurodynamic domains and short-term memory. This indicates the need to improve approaches to the postoperative follow-up management of patients undergoing cardiac surgery, capable of minimizing the development of adverse neurological outcomes.

**Table:** 

Authors' roles & responsibilities	
IVT	Substantial contributions to the acquisition and analysis of data for the work; drafting the work; final approval of the version to be published
OAT	Substantial contributions to the acquisition of data for the work; drafting the work; final approval of the version to be published
IDS	Substantial contributions to the acquisition of data for the work; final approval of the version to be published
OLB	Revising the work critically for important intellectual content; agreement to be accountable for all aspects of the work in ensuring that questions related to the accuracy or integrity of any part of the work are appropriately investigated and resolved; final approval of the version to be published
